# Macroscopic detection of demyelinated lesions in mouse PNS with neutral red dye

**DOI:** 10.1038/s41598-021-96395-4

**Published:** 2021-08-19

**Authors:** Reiji Yamazaki, Yasuyuki Osanai, Tom Kouki, Yoshiaki Shinohara, Jeffrey K. Huang, Nobuhiko Ohno

**Affiliations:** 1 Department of Anatomy, Division of Histology and Cell Biology, School of Medicine, Jichi Medical University, Shimotsuke, Japan; 2 Department of Biology and Center for Cell Reprogramming, Georgetown University, Washington, DC 20057 USA; 3 Division of Ultrastructural Research, National Institute for Physiological Sciences, Okazaki, Japan

**Keywords:** Neuroscience, Neurology

## Abstract

Lysophosphatidylcholine (LPC)-induced demyelination is a versatile animal model that is frequently used to identify and examine molecular pathways of demyelination and remyelination in the central (CNS) and peripheral nervous system (PNS). However, identification of focally demyelinated lesion had been difficult and usually required tissue fixation, sectioning and histological analysis. Recently, a method for labeling and identification of demyelinated lesions in the CNS by intraperitoneal injection of neutral red (NR) dye was developed. However, it remained unknown whether NR can be used to label demyelinated lesions in PNS. In this study, we generated LPC-induced demyelination in sciatic nerve of mice, and demonstrated that the demyelinated lesions at the site of LPC injection were readily detectable at 7 days postlesion (dpl) by macroscopic observation of NR labeling. Moreover, NR staining gradually decreased from 7 to 21 dpl over the course of remyelination. Electron microscopy analysis of NR-labeled sciatic nerves at 7 dpl confirmed demyelination and myelin debris in lesions. Furthermore, fluorescence microscopy showed NR co-labeling with activated macrophages and Schwann cells in the PNS lesions. Together, NR labeling is a straightforward method that allows the macroscopic detection of demyelinated lesions in sciatic nerves after LPC injection.

## Introduction

Demyelinating disorders are characterized by neuropathy associated with the destruction of myelin sheath. Guillain–Barre syndrome and chronic inflammatory demyelinating polyradiculoneuropathy (CIDP) are major PNS demyelinating diseases which are induced by the virus infection or the abnormality of immune system^1,2^. One of the other representative PNS demyelinating diseases is Charcot-Marie-Tooth disease which is induced by the gene mutation including myelin protein zero (P0) and PMP22^3–6^. In the peripheral nervous system (PNS), Schwann cells produce myelin around axons^7,8^. Reprogramming and morphological changes of Schwann cells promote nerve repair or regeneration through the production of neurotrophic factors after peripheral nerve injury^9,10^. The mechanisms of Schwann cells plasticity after nerve injuries have been previously reported. The positive regulator of myelin repair is Notch, Sox2, GPR126, FGFb, and ERK1/2 signaling in Schwann cell^11^. In contrast, HDAC2 is reported as a negative regulator after PNS nerve injury via inhibition of c-Jun and delay the regeneration^12,13^. Transcription factor, such as STAT3 and c-Jun have the role of repair and maintenance for Schwann cells^14^. However, the mechanisms underlying Schwann cell plasticity or remyelination in demyelinating injury and diseases requires further studies, especially in the long terms typically required for regeneration of peripheral nerve neuropathy after PNS demyelination, including Guillain–Barre syndrome and CIDP. While mouse models of experimental demyelination are used to examine the molecular mechanisms and develop the drugs for demyelinating diseases, one of the challenges in the analysis of demyelination models has been the difficulty to identify and selectively acquire tissues of demyelinated lesions for quantitative analysis of transcripts, metabolites, and proteins. Moreover, selecting the demyelinated lesions for electron microscopy (EM), which allows examination of very limited volume of the collected tissues, has also been challenging in morphological investigation of myelin sheaths and evaluation of demyelination and remyelination.

Lysophosphatidycholine (LPC)-induced demyelination is a highly tractable demyelination/remyelination model in mice, in which LPC injection into the white matter of brain and peripheral nerves induces focally demyelinated lesions that spontaneously remyelinate over a reproducible timeframe^15–17^. LPC-induced demyelination is highly informative for evaluation of demyelinated condition and level of remyelination in lesions. Recently, it was shown that the administration of neutral red (NR) into mice allows the macroscopic visualization of demyelinated lesions in freshly dissected CNS tissue^18,19^. NR is a vital dye, which has preference for incorporating into live cells with low intracellular pH, and was previously shown to selectively label macrophages^20,21^. Single intraperitoneal (i.p) injection of NR before sacrifice allowed the detection of demyelinated lesions in the mouse CNS in vivo*.* However, it remained unknown if the NR labeling method would also be applicable to demyelination models of the PNS, and if the tissues with NR labeling could be used for subsequent ultrastructural analyses.

Here, we determined if the single i.p injection of NR can be used to detect demyelinated lesions in LPC-induced demyelination in the mouse PNS. We found that NR could be readily detected macroscopically on site of LPC-injection in sciatic nerves, and EM analysis of NR labeled tissue confirmed that LPC injection resulted in demyelination of sciatic nerve. Moreover, we observed NR co-labeling with activated macrophages and a subpopulation of Schwann cells in lesions. These results suggest that NR injection is a simple and powerful method to detect the lesions in demyelination animal models, and potentially applicable to the wide range of analyses in peripheral demyelinating disease models.

## Results

### Macroscopic detection of neutral red in sciatic nerve demyelination

**Figure 1:**
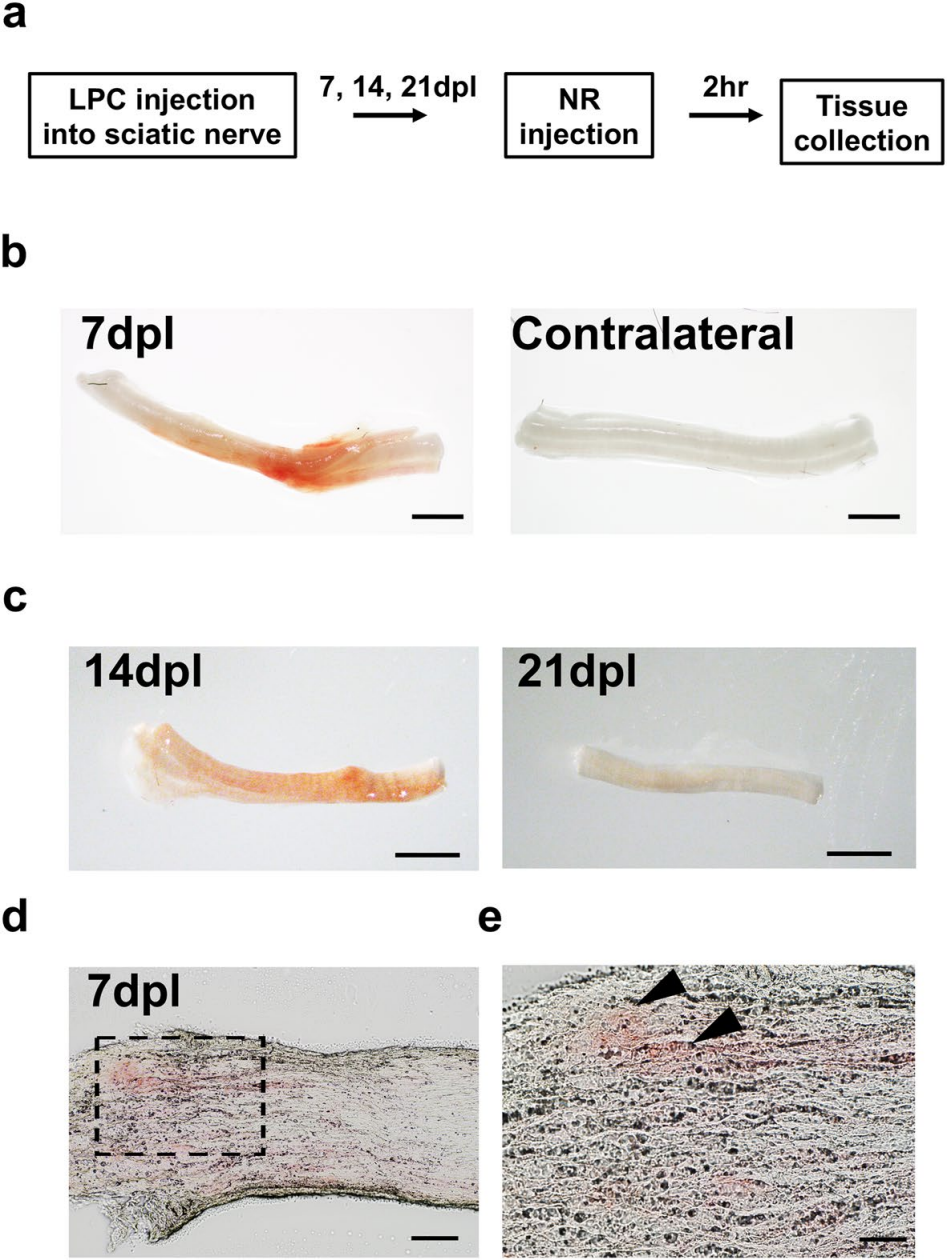
Demyelinated lesion in sciatic nerve identified by neutral red (NR) labeling. (**a**) Experimental design of a sciatic nerve lesion induced by lysophosphatidylcholine (LPC) injection and labeled with NR which was injected 2 h before sacrifice at 7–21 days post lesion (dpl). (**b**) Representative pictures of ipsilateral sciatic nerve with NR labeling and contralateral unlabled nonlesion sciatic nerve at 7 dpl (n = 5 mice per timepoint examined). Scale bar, 1 mm. (**c**) Representative pictures of ipsilateral sciatic nerve with NR labeling at 14 and 21 dpl (n = 3 mice per timepoint examined). Scale bar, 1 mm. (**d**) Longitudinally sectioned nerve showing NR positive areas at 7 dpl. (**e**) Enlarged image of (**d**) (black dotted square). (**b**–**e**) left and right sides of images are proximal and distal sides, respectively. Scale bar, 100 µm (**d**), 50 µm (**e**).

To determine whether NR specifically labels PNS demyelinated lesions, focal demyelination was induced in mouse sciatic nerves by LPC injection. Intraperitoneal (i.p.) injection of NR was performed 2 h before sacrifice of LPC-treated mice. We found some of the mice exhibited a reduction in movement immediately after NR injection, possibly due to the volume of NR received (500 μl). However, these mice recovered within 30 min after injection, and none of the mice displayed any obvious signs of behavioral abnormality throughout the duration of the experiment. To detect NR labeling, LPC injected sciatic nerves and uninjured contralateral nerves were dissected at 7, 14 and 21 days post lesion (dpl) (Fig. 1a), corresponding to timepoints of remyelination^22,23^. We found that NR was readily detectable on the injured sciatic nerve, corresponding to the site of LPC injection at 7 dpl macroscopically or by light microscopy, and no NR labeling was observed on the contralateral nerve (Fig. 1b). Moreover, NR signal decreased at 14 dpl, and was barely detectable at 21 dpl (Fig. 1c). Moreover, longitudinal cryosections of LPC injected sciatic nerve revealed NR had incorporated into the nerve at the site of LPC demyelinated lesion (Fig. 1d,e). However, although NR was observed as a focal lesion on intact LPC-injected sciatic nerves, it displayed non-uniform staining in longitudinal sections. Note that the NR labeling is strongest at the lesion site from which it fades away. These results suggest that NR incorporated into the site of sciatic nerve injury, and the labeling gradually decreased over the course of PNS repair.

## Electron microscopy analysis of NR-labeled lesion

**Figure 2:**
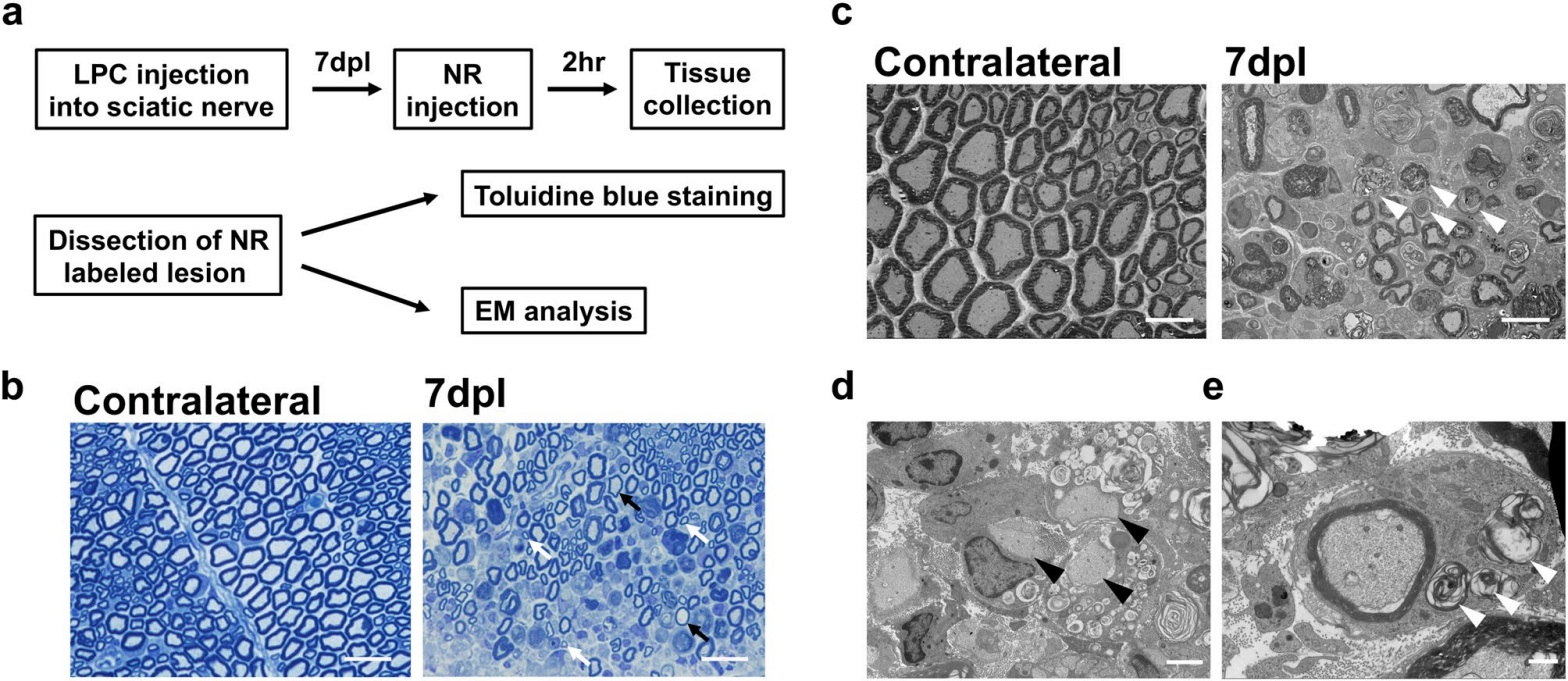
Electron microscopy (EM) analyses following the neutral red labeling. (**a**) Experimental design of EM analyses. Sciatic nerve lesions were induced by lysophosphatidylcholine (LPC) injection and labeled with neutral red (NR) injected 2 h before sacrifice at 7 dpl. Lesions labeled with NR were dissected and embedded in epoxy resin. Semithin sections were stained with toluidine blue. Ultrathin sections were observed by transmission EM. (**b**) Representative images of semithin sections (1 µm) obtained from contralateral and ipsilateral sciatic nerves of the LPC-injected mouse at 7 dpl and stained with toluidine blue. Compared to the contralateral nerve cross-sections, thinner compact myelin (**b**, black arrows) and smaller axons (**b**, white arrows) with myelin ensheathment were present in the LPC-treated nerve. (**c**) In the EM observation, contralateral axons have thick compact myelin, while myelin debris is observed in ipsilateral sciatic nerve at 7 dpl (**c**, white arrowhead). (**d**) Demyelinated axons are observed in the ipsilateral sciatic nerve (**d**, black arrowheads). (**e**) A Schwann cell in ipsilateral sciatic nerve contains myelin debris (**e**, white arrowheads). N = 2 mice examined. Scale bar, 20 µm (**b**), 10 µm (**c**), 2.5 µm (**d**) and 1 µm (**e**).

To assess the identified NR labeled demyelinated lesions, electron microscopy (EM) analysis was performed on NR labeled sciatic nerves at 7 dpl. Transversely sectioned semithin and ultrathin sections were prepared from sciatic nerve tissues following postfixation and embedding into resins, and examined for myelin structure by light microscopic observation with toluidine blue staining and TEM observation (Fig. 2a). Compared with contralateral sciatic nerve semithin sections, the LPC-injected nerves displayed areas lacking compact myelin sheaths, as well as areas with intact myelin sheaths (Fig. 2b). The presence of patches of demyelinated and myelinated axons reflected the non-uniform access of LPC to the sciatic nerve, as was observed with NR labeling in Fig. 1d. TEM analysis of ultrathin sections in NR labeled tissue revealed demyelinated axons as well as myelin debris in lesions (Fig. 2c,d). We also observed the presence of myelin debris within the cytoplasm of some Schwann cells (Fig. 2e), confirming the ongoing process of myelin degradation by Schwann cells^24^. To determine that NR did not contribute directly to demyelination and the extent of myelin debris, we also performed EM analysis on LPC injected sciatic nerves from mice without NR injection at 7 dpl, and found that demyelinated lesion without NR displayed a similar pathology as tissues containing NR labeling (Additional file 1: Fig. S1). These results confirm that NR incorporated into sciatic nerve lesions, and that NR could be used in combination with EM studies for the detection and analysis of demyelinated lesions.

### Fluorescence microscopy analysis localizes NR-labeling in Schwann cells and activated macrophages in demyelinated PNS lesions

**Figure 3:**
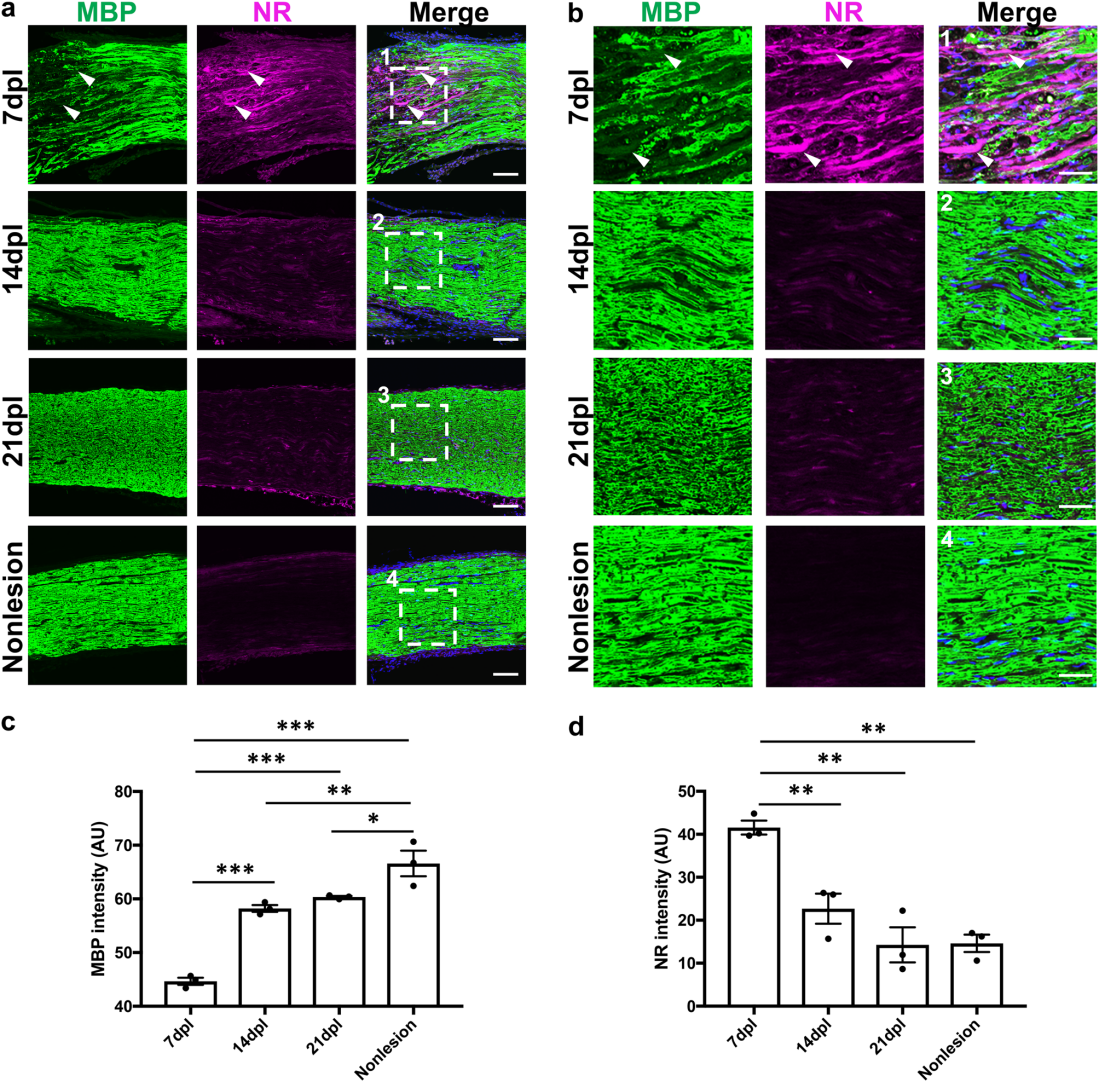
Neutral red (NR) signals were gradually decreased during remyelination. (**a**) Representative confocal images of NR (magenta) and immunofluorescence staining for myelin with anti-MBP (green) in areas with lysophosphatidylcholine injection at 7, 14, 21 dpl and a nonlesion area at 7 dpl (n = 3 mice per timepoint examined). (**b1–4**) Enlarged images of (**a1–4**) (white dotted square). The demyelinated lesion with weak MBP immunoreactivity is shown in 7 dpl image (**a**, **b**; arrowhead). Quantification of fluorescence intensity based on MBP staining (**c**) and NR (**d**) labeling in lesion at 7, 14, and 21 dpl and a nonlesion area at 7 dpl (n = 3). Nuclei were counterstained with Hoechst (blue). Scale bar, 50 µm (**a**) and 20 µm (**b**). Mean ± s.e.m. One-way ANOVA, Tukey–Kramer test. *P < 0.05, **P < 0.01, ***P < 0.001. Graphs were created using Prism 7 (GraphPad Software), https://www.graphpad.com.

NR displays inherent fluorescence with an excitation spectrum of 450-550 nm and a wide emission spectrum with the maximum range 550–650 nm^18^. To detect PNS myelin, lesioned sciatic nerves containing NR labeling were sectioned longitudinally, and immunofluorescence staining for myelin basic protein (MBP) was performed. We observed strong NR fluorescence in areas containing low MBP expression, corresponding to areas of demyelination, at 7 dpl (Fig. 3a,b). Furthermore, quantitative analysis demonstrated that the NR signal significantly decreased as immunoreactivity of MBP increased at 14 dpl, and became indistinguishable from uninjured tissue by 21 dpl when the MBP staining was restored (Fig. 3c,d). However, NR signals might be sometimes visible in what appears to be pericytes and tissue resident macrophages in non-lesioned areas. (see Fig. 5). These results demonstrate that NR incorporation into demyelinated lesions in sciatic nerve could be observed by fluorescence microscopy.

**Figure 4:**
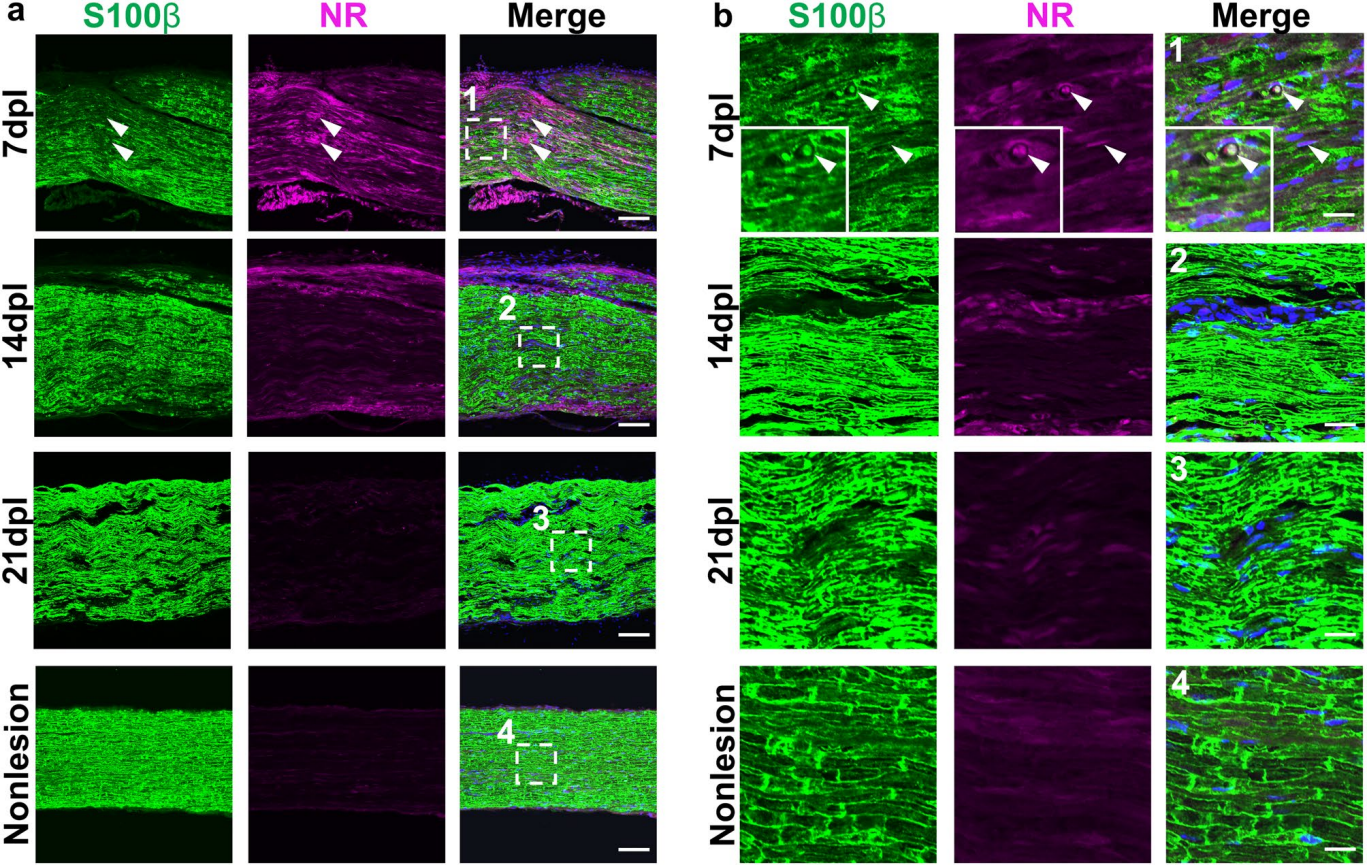
Neutral red (NR) colocalizes with Schwann cells in demyelinated lesions. (**a**) Representative confocal images of NR (magenta) and immunofluorescence staining for Schwann cell labeled with anti-S100β (green) in lysophosphatidylcholine (LPC)-treated nerves (7, 14, and 21 dpl) or non-lesioned nerves (n = 3 mice examined per timepoint). The demyelinated lesion is shown in 7 dpl image (arrowhead). (**b1–4**) Enlarged images of (**a1–4**) (white dotted square). Highly magnified images show NR signal were partially colocalized with S100β-positive cells (arrowhead). *Insets* Enlargements of double-positive cell. Nuclei were counterstained with Hoechst (blue). Scale bar, 50 µm (**a**) and 20 µm (**b**).

To examine Schwann cell distribution in NR labeled lesions, S100β^24^ immunostaining was next performed. We observed decreased S100β labeled cells in areas of high NR labeling, compared to areas around the NR-labeled lesions and in non-lesion areas at 7 dpl (Fig. 4a). Some of NR signals were detected inside S100β-positive Schwann cells within the lesion, and no NR signals were detectable in S100β-positive Schwann cells in non-lesioned areas (Fig. 4b). Similar to MBP staining, NR signals decreased as immunoreactivity of S100β increased at 14 dpl, and the lesioned tissue was indistinguishable from uninjured tissue by 21 dpl (Fig. 4a,b). These results demonstrate that NR labeled demyelinated lesion exhibits decreased Schwann cells at 7 dpl that gradually repopulates back to the lesion by 21 dpl. Moreover, Schwann cells containing NR labeling in lesions at 7 dpl likely correspond to phagocytosing Schwann cells as was observed by EM.

**Figure 5:**
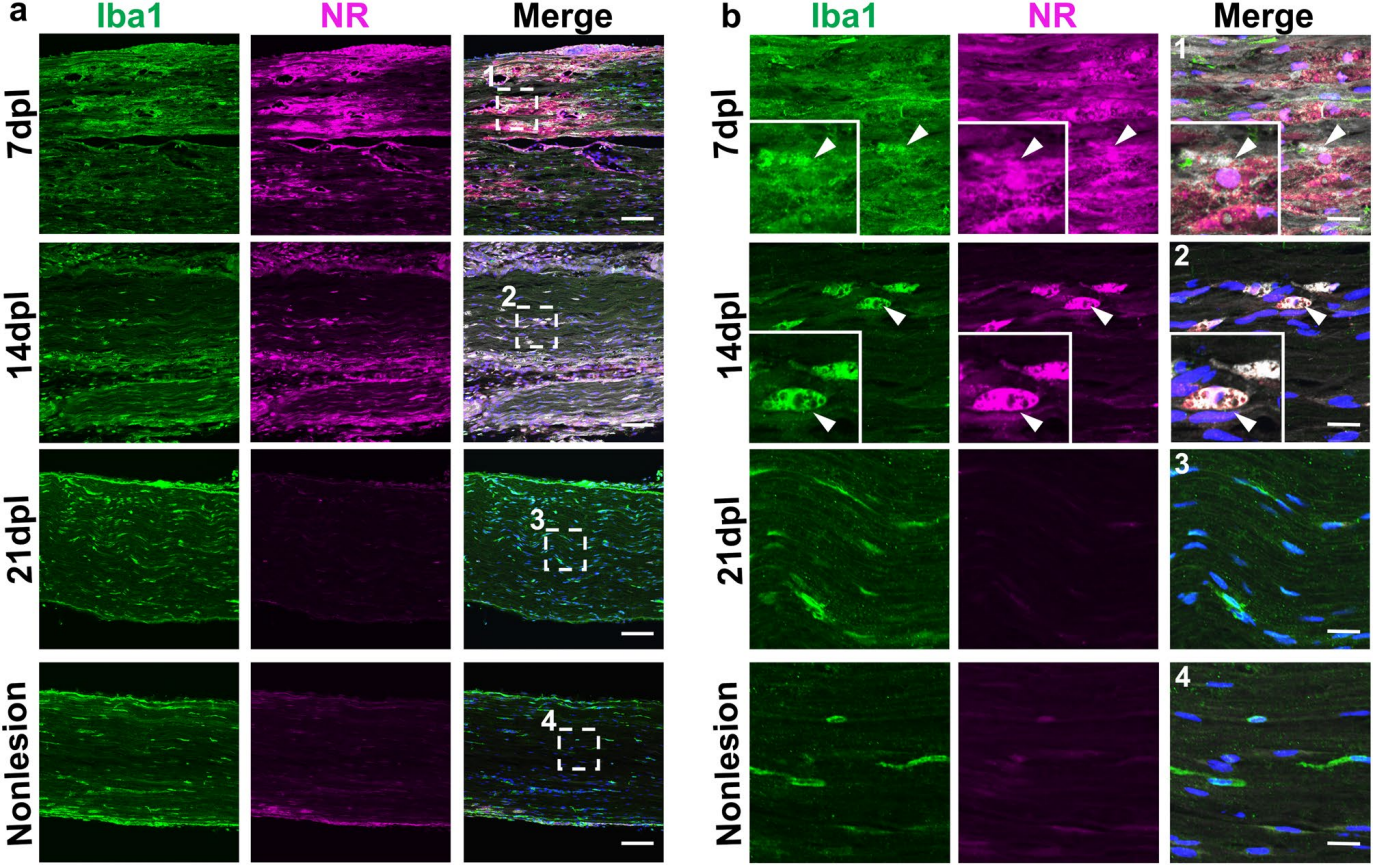
Neutral red (NR) colocalized with Iba1-positive macrophages in demyelinated lesions. (**a**) Representative confocal images of NR (magenta) and immunofluorescence staining for macrophages labeled with anti-Iba1 (green) at low (**a**) and high (**b**) magnification in areas with lysophosphatidylcholine injection at 7, 14 and 21 dpl and a nonlesion area at 7 dpl (n = 3 mice per timepoint examined). (**b1–4**) Enlarged images of (**a1–4**) (white dotted square). Iba1-positive macrophages colocalized with NR (arrowhead). *Insets* Enlargements of double-positive cells. No NR labeling was detected in Iba1-positive macrophages in nonlesion area. Scale bar, 50 µm (**a**) and 20 µm (**b**). Nuclei were counterstained with Hoechst (blue).

Next, we examined macrophage distribution on NR labeled tissues by immunostaining for Iba1 expression, a pan-macrophage marker. We found that Iba1-positive macrophages accumulated in lesions at 7 dpl and strongly co-labeled with NR (Fig. 5a,b). Moreover, NR intensity decreased with reduction of Iba1-positive macrophages between 7 and 21 dpl (Fig. 5a,b). In contrast, NR labeling was weakly detected or undetectable in Iba1-positive macrophages in non-lesioned peripheral nerve at 7 dpl (Fig. 5a,b), suggesting NR did not incorporate into resting macrophages.

**Figure 6:**
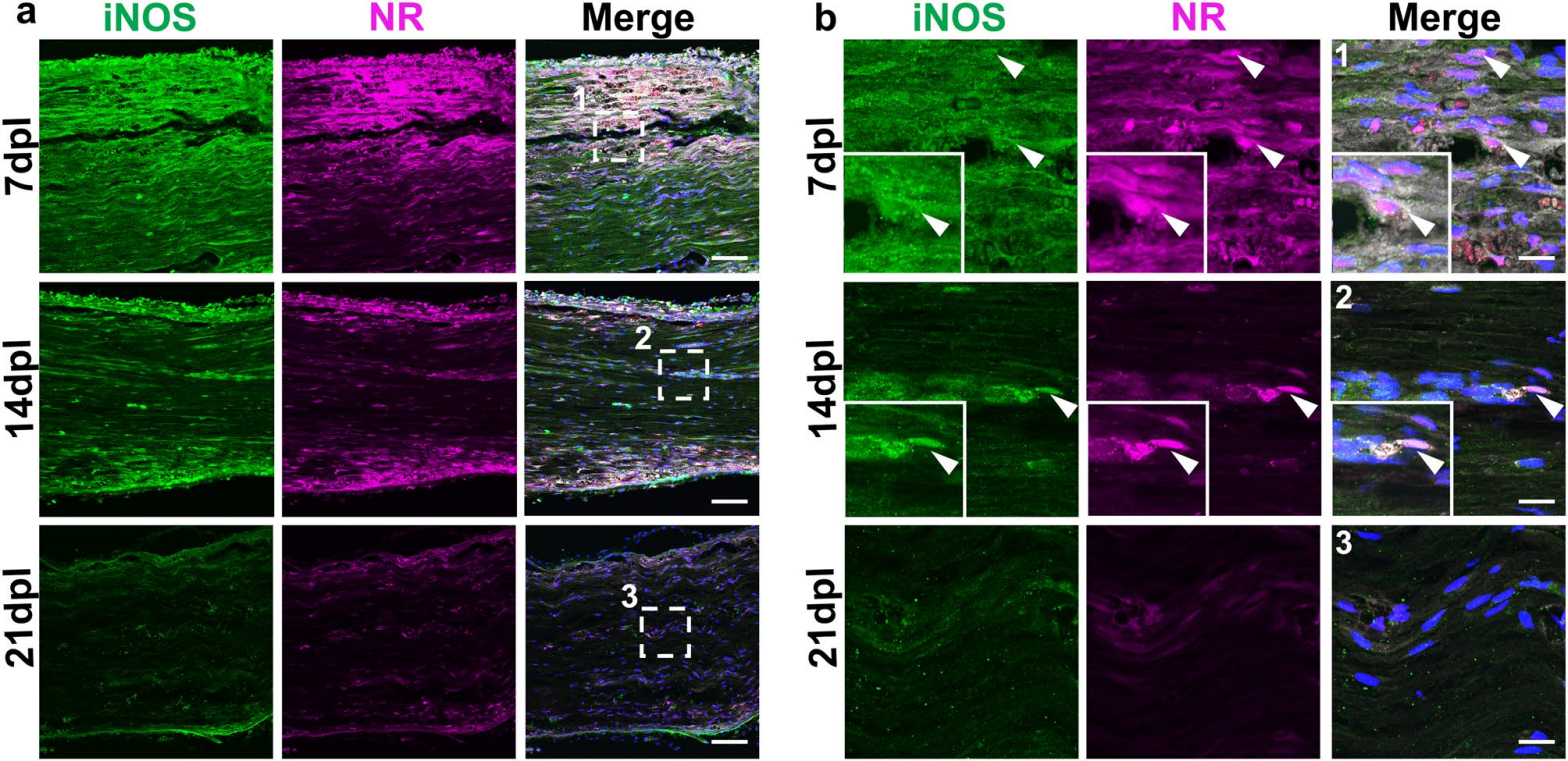
Neutral red (NR) colocalized with M1 type macrophages. Representative confocal images of NR (magenta) and immunofluorescence staining for iNOS (green) at low (**a**) and high (**b**) magnification in areas with lysophosphatidylcholine injection at 7, 14 and 21 dpl (n = 3 mice per timepoint examined). (**b1–3**) Enlarged images of (**a1–3**) (white dotted square). iNOS-positive cells colocalized with NR at 7 dpl (**b**, arrowhead). iNOS positive cells with NR signals are also present at 14 dpl. *Insets* Enlargements of double-positive cell. Nuclei were counterstained with Hoechst (blue). Scale bar, 50 µm (**a**) and 20 µm (**b**).

**Figure 7:**
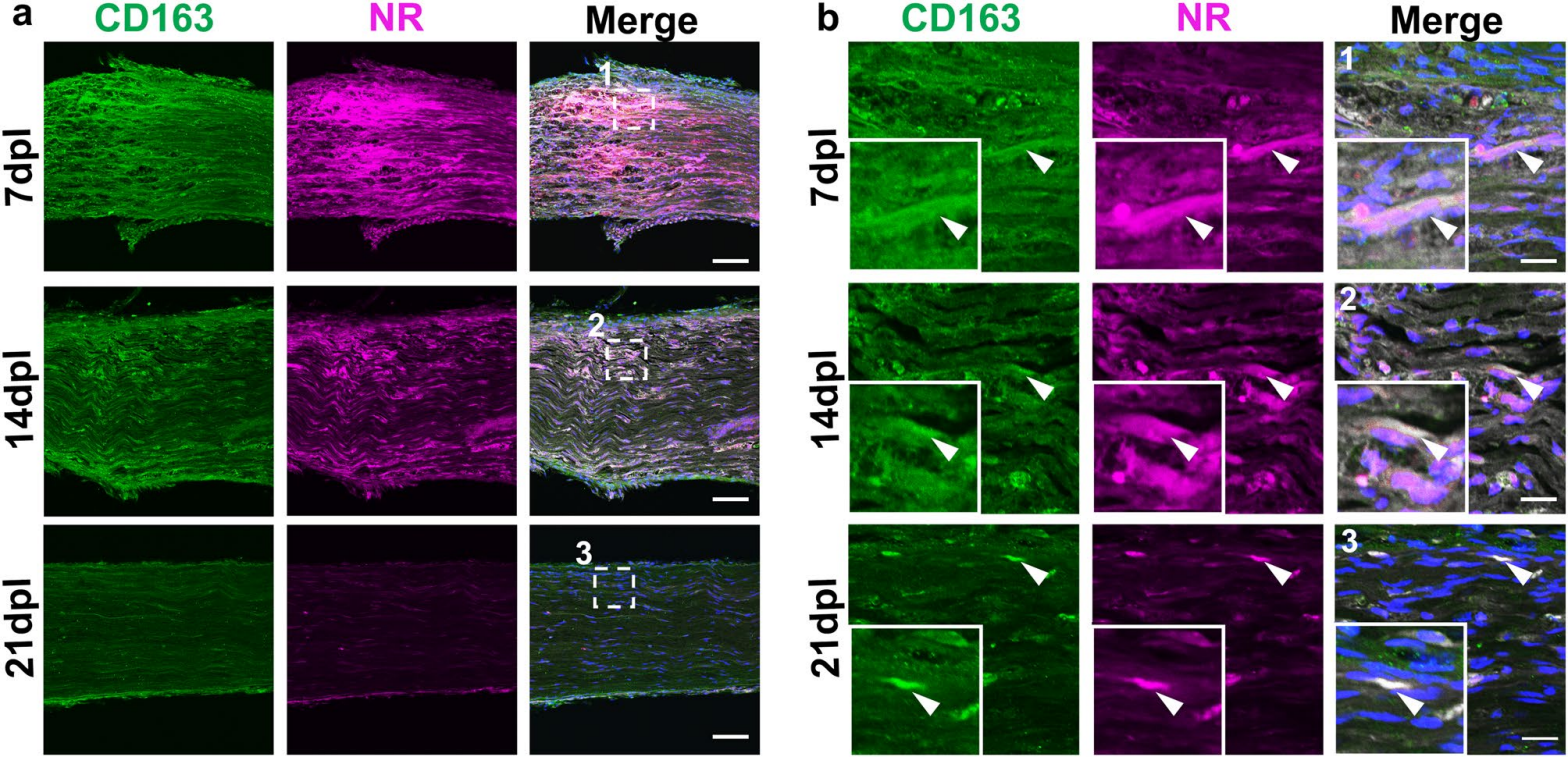
Neutral red (NR) colocalized with CD163-positive M2 type macrophages in demyelinated lesions. (**a**) Representative confocal images of NR (magenta) and immunofluorescence staining for macrophages labeled with anti-CD163 (green) at low (**a**) and high (**b**) magnification in areas with lysophosphatidylcholine injection at 7, 14 and 21 dpl (n = 3 mice per timepoint examined). (**b1–3**) Enlarged images of (**a1–3**) (white dotted square). CD163-positive cells colocalized with neutral red at each timepoint (**b**, arrowhead). *Insets* Enlargements of double-positive cell. Nuclei were counterstained with Hoechst (blue). Scale bar, 50 µm (**a**) and 20 µm (**b**).

Macrophages under injury exhibit a spectrum of activation states, which includes pro-inflammatory M1-like and anti-inflammatory M2-like macrophages having distinctive gene expression patterns^25^. To determine whether NR labels M1-like or M2-like macrophages, we performed immunofluorescence staining using antibodies against iNOS, a pro-inflammatory macrophage marker (Fig. 6a,b), and CD163, an anti-inflammatory marker (Fig. 7a,b). We observed strong NR signals in both iNOS-positive and CD163-positive cells at 7 dpl, and the corresponding decrease of iNOS and CD163-positive cells with NR labeling in lesions by 21 dpl (Figs. 6b, 7b). These results suggest that NR dye labeled both M1 and M2 like macrophages in demyelinated lesion, and that NR labeling gradually decreased with the reduction of inflammation during remyelination.

### NR labeling colocalizes with lysosomes

**Figure 8:**
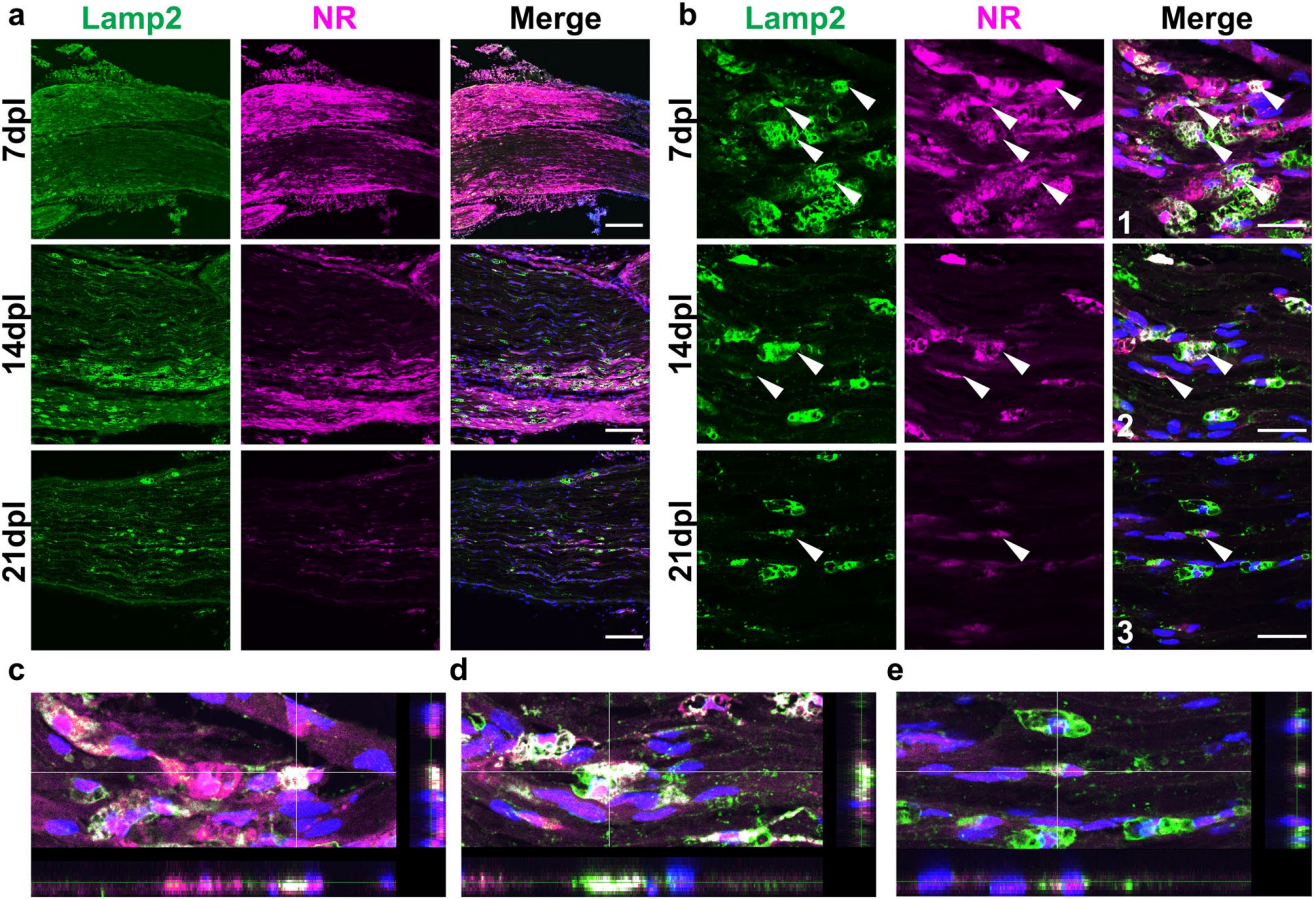
Neutral red (NR) colocalized with Lamp2-positive lysosome of cells in demyelinated lesions. (**a**) Representative confocal images of NR (magenta) and immunofluorescence staining for lysosomes labeled with anti-Lamp2 (green) at low (**a**) and high (**b**) magnification in areas with lysophosphatidylcholine injection at 7, 14 and 21  dpl (n = 3 mice per timepoint examined). Lamp2-positive lysosome colocalized with neutral red at 7 (**c**), 14 (**d**) and 21 (**e**) dpl (**b**, arrowhead). Overlay of z-stack images with vertical (right) and horizontal (bottom) cross-sections are shown (**b1–3**: z-stack images of Merge). Nuclei were counterstained with Hoechst (blue). Scale bar, 50 µm (**a**) and 20 µm (**b**). z-stack images were analysed using Olympus Fluoview FV-ASW version 1.7, https://www.olympus-lifescience.com.

Previously, it has been reported that NR incorporates into the lysosomes of live cells^20,21^. The detection of activated macrophages and phagocytic Schwann cells in lesions suggest these cells display high lysosomal content. To examine the intracellular localization of NR in the demyelinated lesion, we performed immunostaining using Lamp2 antibody as a lysosomal marker (Fig. 8a). Lamp2-positive signals were colocalized with NR signals in the lesion at 7 and 14 dpl (Fig. 8b). Both Lamp2 and NR signals were gradually decreased during remyelination. Furthermore, we also examined colocalization by 3D analysis. NR signals were colocalized with Lamp2-positive lysosomes in z-stack images (Fig. 8c–e: Movies S1–S3). These results suggest NR has a preference for cellular compartment with high lysosomal activity in the demyelinated lesion.

## Discussion

In this study, we determined if i.p injection of NR can be used to detect demyelinated lesions in the PNS after LPC injection. We found that NR readily labels demyelinated sciatic nerve, and that NR labeling diminishes following remyelination. We detected NR incorporation in activated macrophages and phagocytic Schwann cells in demyelinated lesions, and the decrease of NR labeling coincided with decrease of the activated macrophages. Furthermore, we found that NR labeled tissue could be successfully applied for EM analysis of demyelinated axons and myelin debris in lesions. Since demyelinated lesions could be readily identified macroscopically, NR labeling facilitates the straightforward acquisition of small tissue pieces containing a focal demyelinated lesion for subsequent sample preparation and EM analysis.

Our data indicated that activated macrophages along with significant myelin loss were observed in NR-labeled lesions, and NR-labeling was detected in both iNOS-positive (M1-like) and CD163-positive (M2-like) macrophages as well as S100β-positive Schwann cells. In PNS demyelinated lesions, Schwann cells, monocytes or hematogenous, endogenous or mesenchymal cells have been reported to display phagocytic activity^24,26,27^. Our data showing S100β-positive cells with NR-labeling at 7 dpl suggest that NR incorporates into phagocytotic Schwann cells in lesions. Moreover, the decreased NR labeling at 21 dpl coincided with the reduction of macrophage activity and myelin repair. These results suggest that the NR labeling method is also applicable to visualize damaged regions in PNS demyelination or injury.

While it remained to be elucidated if NR labeling can be applied to demyelination models other than LPC injection, the macroscopic identification of demyelinated PNS lesions by NR labeling would be useful to reduce the error in lesion detection and facilitate subsequent experimental analyses to elucidate molecular mechanisms of pathology in demyelination as well as recovery through remyelination. Indeed, previous study has reported that NR labeling could be used for RT-PCR, Western blot and flow cytometry analysis of unfixed spinal cord demyelinated lesions^18^.

Although fluorescence microscopy can be applied to examine NR incorporated cells in lesions and the strong NR staining was observed in areas of demyelination, we have also observed fluorescent NR labeling outside of the lesion site. It is possible that NR is also incorporated into activated macrophages or other phagocytic cells outside of lesions. Moreover, a caveat with fluorescence imaging is that weak NR-positive signals, due to the wide emission fluorescence spectrum of NR, could also be observed extracellularly, and autofluorescence in the perineurium or epineurium containing connective and adipose tissue surrounding the sciatic nerve^28,29^ may also be detected under fluorescence microscopy even after destaining with 50% ethanol/1% glacial acetic acid. On the other hand, since demyelinated fibers are non-uniformly distributed in LPC injected sciatic nerves, inhomogeneous NR labeling might be detected at the lesioned tissue. Moreover, lactic acidosis is characteristic for inflammatory processes^30^. Therefore, the extracellular NR signals could represent general low pH within the microenvironment of the nerve lesion. While combinations of these reasons might be responsible for the diffuse NR labeling, optimization of the tissue processing procedure and the staining procedure may improve background staining under fluorescence microscopy for future studies.

In summary, our results suggest that NR labeling is a tractable method for evaluation of demyelinated state and recovery from demyelination in PNS tissues. The method may be used to identify the molecular mechanisms of Schwann cell remyelination, and applied to investigate drug targets or future drug screening for PNS repair.

## Methods

### Animals

10–12 week old male C57BL/6J mice (20–30 g) were purchased from Japan SLC or Clea Japan. These mice were kept in standard cages with fewer than 5 mice per cage at 20–25 °C on a 12 h light/dark cycle. All animal experiments were performed in accordance with the ARRIVE (Animal Research: Reporting In Vivo Experiments) guidelines. All efforts were made to use only the number of animals necessary. All experiments were approved by the Institutional Animal Care and Use Committee (IACUC) at Jichi Medical University (approval number 19034-03) and performed in accordance with the guidelines on the care and use of animals of this committee.

### Focal demyelination of the sciatic nerve and NR injection

Demyelination was induced by injection of 1% LPC (Sigma-Aldrich) in phosphate buffered saline (PBS) into sciatic nerve^31^. Mice were anesthetized by the mixture of 3 drugs (0.3 mg/kg of medetomidine, 4.0 mg/kg of midazolam, and 5.0 mg/kg of butorphanol) and the right sciatic nerve was exposed. Hamilton syringe (31 gauge) connected to the micro injector (IMS-3; Narishige) and manipulator (YOU-1; Narishige) was inserted into the nerve and kept for 3 min before injection of LPC. Sciatic nerve was injected with 1 µl of 1% LPC using the micro injector, and the needle was kept for 5 min after injection to reduce leak along the needle track. The mice were sacrificed at 7, 14 or 21 days post lesion (dpl). Prior to the sacrifice, 500 µl of 1% NR (10 mg/ml; Sigma-Aldrich) in PBS was injected by intraperitoneal (i.p) injection for each mouse as described previously^18,19^.

### Tissue processing and staining procedures

Two hours after the NR injection, mice were transcardially perfused with 1 ml/g body weight of PBS. Sciatic nerves were rapidly dissected and immersed overnight in 4% (w/v) paraformaldehyde (PFA) in PBS at 4 °C for fixation. Fixed sciatic nerves were sequentially immersed overnight in 15% and 30% (w/v) sucrose (Fujifilm Wako Pure Chemical Co.) in PBS at 4 °C for cryoprotection, and frozen in OCT compound (Sakura Finetec). Cryosections (10 µm thick) were prepared using a cryostat (CM3050; Leica Microsystems), collected on coated glass slides (Matsunami), and stored at – 30 °C. Immunofluorescence staining of cryosections were performed according to a previously described protocol with slight modifications^32^. Before immunofluorescence staining, cryosections were incubated with a destain solution of 50% ethanol/1% glacial acetic acid to reduce the background for 30 min^33^. For immunofluorescence staining, cryosections were permeabilized and blocked in blocking buffer (0.3% TX-Tris-Buffered Saline (TBS) and 10% normal goat serum) for 1 h. Then the sections were incubated overnight at 4˚C with primary antibodies in the blocking buffer. Thereafter, cryosections were incubated at RT for 1 more hour, washed in 0.3% TX-TBS, incubated for 3 h at RT with secondary antibodies in 0.3% TX-TBS, washed in 0.3% TX-TBS, and embedded in Fluoromount-G (Southern Biotech)^32^. Some other cryosections were stained with 1% toluidine blue. The stained sections were observed by light microscopy (BX63; Olympus) or confocal microscopy (FV1000; Olympus).

### Antibodies

The primary antibodies for immunofluorescence staining were rabbit polyclonal anti-ionized calcium binding adapter molecule 1 (Iba1) (1:100; Fujifilm Wako Pure Chemical Co.), rabbit polyclonal anti-S100β (1:1000; abcam), rabbit polyclonal anti-CD163 (1:100; Bioss), rabbit polyclonal anti-inducible nitric oxide synthase (iNOS) (1:50; BD Biosciences), rat monoclonal anti-myelin basic protein (MBP, aa-82-87) (1:100; AbD Serotec), rat monoclonal anti-Lamp2 (1;100; abcam). Alexa Fluor 488-conjugated species-specific secondary antibodies (1:500; Thermo Fisher Scientific) with 1 µg/ml Hoechest 33342 (Thermo Fisher Scientific) for labeling nuclei.

### EM analysis

EM analysis was performed as previously reported^34^. Two hours after the NR injection, anesthetized adult mice were transcardially perfused with PBS for a short period of time and subsequently with 4% PFA and 2.5% glutaraldehyde in 0.1 M PB (pH 7.4). Sciatic nerve tissues were immersed overnight in the same fixative at 4 °C for post fixation of tissue after perfusion. For transmission electron microscopy (TEM), these samples were post-fixed in cold 2% OsO_4_ in 0.1 M PB for 90 min, dehydrated in a graded ethanol series and embedded in Quetol 812 epoxy resin (Nisshin EM Co.). The resin was incubated at 60 °C for two nights to ensure polymerization. Prior to TEM observation, semithin sections were cut at 1 µm thick, stained with 1% toluidine blue and examined under a light microscope (BX63; Olympus). Ultrathin sections (70 nm thick) were prepared with an ultamicrotome (Ultracut UCT, LEICA) and stained with uranyl acetate and lead citrate. The ultrathin sections were observed by TEM (HT7700; Hitachi High-Tech).

## Statistical analysis

Quantitative analysis is presented as mean ± standard error of the mean (s.e.m) from each image. For quantification, the images of areas with LPC injection and fluorescent intensities of the images captured by confocal microscopy were determined by Fiji-imageJ. Note that the images of different conditions (7, 14, 21 dpl and non lesioned) have been obtained using the same image acquisition settings for quantitative analysis. The intensities of the positive signals were measured per unit areas and compared in arbitrary units (AU). Statistical analyses were performed using Prism 7 (GraphPad Software). Statistical comparisons were performed using one-way ANOVA, followed by a Tukey Kramer test. The significance was indicated as a P value: *P < 0.05, **P < 0.01, ***P < 0.001.

## Supplementary Information

Supplementary Information 1.

Supplementary Video 1.

Supplementary Video 2.

Supplementary Video 3.

## Data Availability

The datasets used and/or analysed during the current study available from the corresponding author on reasonable request.

## Abbreviations

dpl: Days post lesion
CIDP: Chronic inflammatory demyelinating polyradiculoneuropathy
EM: Electron microscopy
Iba1: Inonized calcium binding adapter molecule
iNOS: Inducible nitric oxide synthase
i.p.: Intraperitoneally
LPC: Lysophosphatidylcholine
MBP: Myelin basic protein
NR: Neutral red
TEM: Transmission electron microscopy
