# Supplementary material for: Macroscopic detection of demyelinated lesions in mouse PNS with neutral red dye
Source: Sci Rep. 2021 Aug 19;11:16906. doi: 10.1038/s41598-021-96395-4 (PMC8377033; doi:10.1038/s41598-021-96395-4)
Supplement: Supplementary file 1 — Supplementary Information 1. [file 41598_2021_96395_MOESM1_ESM.docx]

**Macroscopic detection of demyelinated lesions in mouse PNS with neutral red dye**

Reiji Yamazaki^1*^, Yasuyuki Osanai^1^, Tom Kouki^1^, Yoshiaki Shinohara^1^, Jeffrey K. Huang^2^, Nobuhiko Ohno^1, 3^

^1^Department of Anatomy, Division of Histology and Cell Biology, School of Medicine, Jichi Medical University, Shimotsuke, Japan

^2^Department of Biology and Center for Cell Reprogramming, Georgetown University, Washington, DC 20057, USA

^3^Division of Ultrastructural Research, National Institute for Physiological Sciences, Okazaki, Japan

^*^Correspondence should be addressed to R.Y. ([ryamazaki@jichi.ac.jp](mailto:ryamazaki@jichi.ac.jp))


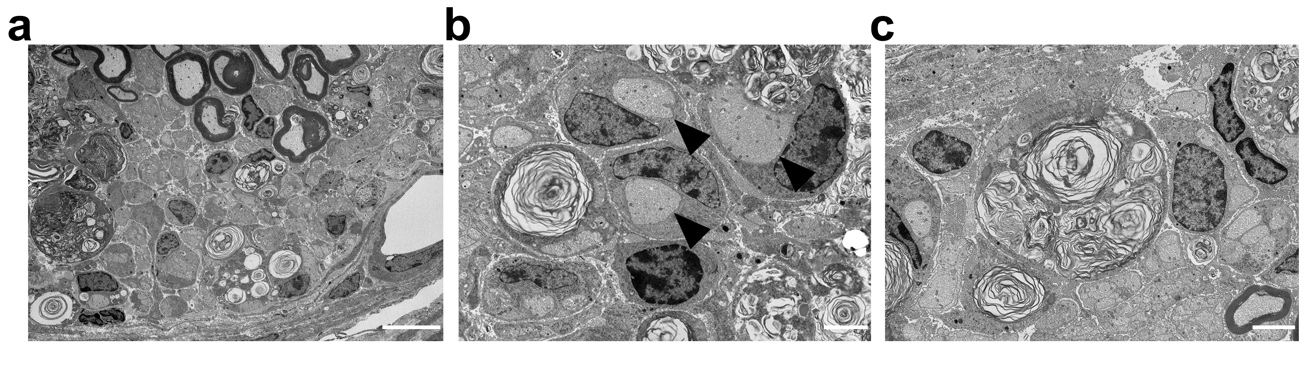


**Supplementary Figure S1. Electron microscopy (EM) analyses without neutral red.** (**a**) Representative images of ultrathin section obtained from sciatic nerve of lysophosphatidylcholine-injected mouse at 7dpl without neutral red. (**b**) Demyelinated axons are observed in the ipsilateral sciatic nerve (**b**, black arrowheads). (**c**) myelin debris is observed in sciatic nerve at 7dpl. Scale bar, 10 µm (**a**), 2.5 µm (**b**) and 1 µm (**c**).

**Supplementary movies**

**Movie S1.** Representative movie of the colocalization with Lamp2 and NR at 7dpl.

**Movie S2.** Representative movie of the colocalization with Lamp2 and NR at 14dpl.

**Movie S3.** Representative movie of the colocalization with Lamp2 and NR at 21dpl.
